# NRF2: Master regulator of cellular homeostasis and therapeutic vulnerability in cancer

**DOI:** 10.1016/j.redox.2026.104050

**Published:** 2026-01-23

**Authors:** Wei-tai Chen, Nicholas W. McKee, Damaris Kuhnell, Matthew Dodson

**Affiliations:** a Department of Pharmacology and Toxicology, College of Pharmacy, University of Arizona, Tucson, AZ, 85721, USA; b Department of Environmental and Public Health Sciences, College of Medicine, University of Cincinnati, Cincinnati, OH, 45267, USA

**Keywords:** Nrf2, Keap1, Antioxidant response, Metabolism, Proteostasis, Toxicology, Oxidative stress, Therapeutics, Cancer

## Abstract

The transcription factor nuclear factor erythroid 2-related factor 2 (NRF2) is best known for its regulation of the antioxidant response. However, its mediation of other pathways, including key aspects of metabolic and protein homeostasis, has continued to emerge. Accompanying this emergence is an evolved understanding that NRF2 induction across different disease contexts can be beneficial or detrimental depending on the length of activation. This has played an important role in progressing the field forward, as inducing NRF2 is not always the best course of action, and inhibition has gained traction as a viable strategy for treating cancer and other pathologies where NRF2 is chronically active. Despite its rapid growth and a wealth of experimental promise, a persistent shortcoming in the field is a lack of NRF2-specific therapeutics used in clinic. Thus, despite recent advances, there is still room for progress in translating experimental evidence into therapeutic reality. In this review, we will provide a summary of NRF2 regulation and an update on its expanded network of downstream transcriptional programs. We will also discuss targeting NRF2 in disease, focusing on intervention versus prevention depending on the pathological context. Finally, we will briefly highlight current limitations in the field, as well as ongoing approaches that show promise for finally targeting this critical cascade in patient populations.

## 1 Introduction

An organism's ability to survive depends on its capacity to successfully mitigate stress. Over time, complex, interconnected signaling networks have evolved to resolve different forms of stress and restore proper homeostasis. Importantly, the nature, level, and timing of the response is dictated by the stressor, as distinct downstream signaling cascades are activated to initiate the appropriate response. This is particularly important in the context of acute, cytotoxic stressors, where rapid and targeted activation of a robust response is designed to preserve viability and prevent initiation of pro-death cascades. One master regulator of cellular homeostasis, particularly in response to exogenous insult, is nuclear factor erythroid 2-related factor 2 (NRF2), a transcription factor that mediates the expression of antioxidant response element (ARE)-containing genes. Since its discovery in the mid-1990s, our understanding of NRF2 regulation and function has continued to progress at an exponential rate, starting with the early investigations into its mediation of xenobiotic detoxification and the antioxidant response, to more recent findings showing it dictates key aspects of iron homeostasis, as well as lipid and carbohydrate metabolism [1].

Along with the discovery of novel NRF2-regulated transcriptional programs, the pathological relevance of the NRF2 pathway has also continued to expand, ranging from cancer, diabetes, and neurodegeneration to fatty liver, chronic kidney, and cardiovascular disease [2]. This continued expansion of NRF2 importance across a diverse array of disease states has led to an evolved understanding that it can play a positive or negative role depending on the pathological context. Along these lines, whether NRF2 activation is beneficial or detrimental is primarily determined by the mode and length of its activation, with acute, regulated induction being protective, and a prolonged, dysregulated response being pathogenic. Accordingly, pharmacological induction or inhibition of this key transcription factor, as well as its up- or downstream mediators, continues to represent an active area of interest. However, despite a vast body of scientific literature indicating the therapeutic promise of targeting NRF2 to treat disease, several key hurdles remain, and very few NRF2-based therapeutics have progressed to clinical trials. This is due, in large part, to the off-target effects associated with most of the best candidates identified to date. Compounding the problem, NRF2 itself can be regulated at the epigenetic, transcriptional, pre-translational, and post-translational levels by a variety of mechanisms, and several binding partners have been identified that regulate NRF2 stability and function [1]. Thus, as our understanding of the intricacies of NRF2 regulation, including what downstream targets it directly or indirectly regulates across different pathological states, continues to advance, targeting this critical cascade should progress from therapeutic promise to clinical reality.

In this review, we will outline what is currently known regarding mechanisms of NRF2 regulation, including modes of activation and inhibition, crosstalk with other stress response pathways, and the transcriptional programs that it governs. We will then provide a detailed description of the stressors where NRF2 activation or inhibition have been shown to affect the phenotype, emphasizing the models, compounds, and pathological settings with the most translational relevance. Finally, we will briefly discuss what the future of NRF2 holds, including current limitations and the ongoing efforts to push the field forward.

## 2 The NRF2 signaling pathway

### 2.1 Overview

**Fig. 1:**
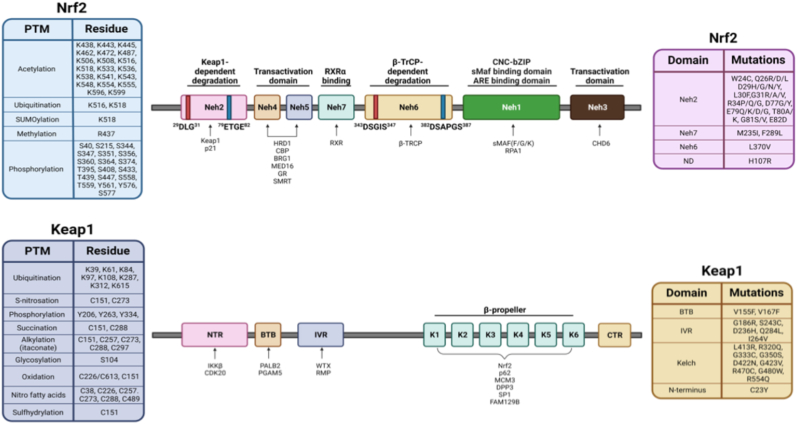
**Domain architecture, post-translational modifications, and disease-associated mutations of NRF2 and KEAP1.** Schematic representation of the human NRF2 (*NFE2L2*) and KEAP1 proteins highlighting functional domains, regulatory interactions, post-translational modification (PTM) sites, and reported mutations. **Top:** NRF2 is shown with its seven conserved Neh domains. The Neh2 domain contains the DLG and ETGE motifs required for KEAP1-dependent ubiquitination and proteasomal degradation. Neh4 and Neh5 function as transactivation domains and mediate interactions with transcriptional coactivators including CBP/p300 and related complexes, whereas Neh7 mediates repression through interaction with RXRα. Neh6 contributes to KEAP1-independent, β-TrCP-mediated degradation, and the Neh1 domain contains the CNC-bZIP region required for small MAF heterodimerization and antioxidant response element (ARE) binding. Neh3 supports transcriptional activation via interactions with chromatin-associated factors. Major sites of acetylation, ubiquitination, SUMOylation, methylation, and phosphorylation are indicated, along with representative disease-associated mutations mapped to specific domains. **Bottom:** KEAP1 is depicted with its N-terminal region (NTR), BTB domain (required for homodimerization and CUL3 binding), intervening region (IVR), and C-terminal Kelch β-propeller domain (K1–K6 repeats) responsible for binding NRF2. Key cysteine residues subject to redox-sensitive modifications (including alkylation, oxidation, nitrosylation, and succination) are highlighted, reflecting KEAP1's role as a redox sensor. Post-translational modifications and representative mutations across KEAP1 domains are indicated, illustrating mechanisms by which redox signaling, genetic alterations, or covalent modification disrupt KEAP1-NRF2 regulation.

NRF2, along with NRF1, NRF3, NF-E2, Bach1, and Bach 2, is a member of the cap'n’collar basic leucine zipper (CNC-bZIP) family of transcription factors. Structurally, NRF2 is comprised of 7 Neh domains, each with its own specialized purpose for dictating NRF2 function and stability (Fig. 1). While NRF2 has been linked to the activation of hundreds of putative target genes, those that have been fully validated (i.e., contain a ChIP-seq-, deletion construct-, or mutation-verified functional ARE) number closer to 50. Importantly, one continuing limitation in the field is that many studies correlate changes in putative target gene expression with NRF2 induction or inhibition without verifying that NRF2 binds to an ARE, or that mutation of the ARE can reverse the phenotype. This is a critical step, as the pleiotropic nature of NRF2 signaling can result in indirect activation or suppression of a multitude of pathways. Thus, careful consideration must be taken in determining if an NRF2 dictated response is direct or indirect. With regards to the pathways it governs, NRF2 target genes have been implicated in almost every aspect of cellular function, including glutathione synthesis, xenobiotic/drug detoxification, iron homeostasis, DNA repair, carbohydrate and lipid metabolism, proteasome assembly, and autophagy [1]. As one might expect based on the diverse array of pathways mediated by NRF2 targets, dysregulation of the NRF2 cascade plays a critical role in disease progression, and a great deal of scientific effort continues to be directed towards successfully targeting this pathway across a variety of disease states.

### 2.2 NRF2 degradation machinery

While NRF2 expression can be regulated at the DNA and mRNA levels, it is mainly controlled at the protein level by three critical E3 degradation complexes: Kelch-like ECH associated protein 1-Cullin3-Ring box 1 (Keap1-Cul3-Rbx1), S-phase kinase-associated protein 1-Cullin1-Rbx1/β-transducin repeat-containing protein (SCF-β-TRCP), and HRD1 (also known as synoviolin). Interestingly, each of these complexes primarily operates in a different subcellular compartment, i.e., the cytosol, nucleus, and endoplasmic reticulum, respectively, and despite having the same goal of targeting NRF2 for degradation, accomplish this task via differing mechanisms. Importantly, this diversity in localization and machinery capable of degrading NRF2 not only indicates an evolved need for tight regulation, but also an ability to respond to a diverse array of stressors, regardless of the source or subcellular localization. Key aspects of each degradation complex, including mechanisms whereby their inhibition leads to activation of the NRF2 cascade, will be discussed below.

#### 2.2.1 Keap1-Cul3-Rbx1

Perhaps the most prevalent and well-studied suppressor of NRF2 at the protein level is Keap1, originally identified by Dr. Masayuki Yamamoto's group in the late 1990s [3]. Keap1 consists of an N- and C-terminal region, a Broad complex, Tramtrack and Bric-à-Brac (BTB) domain, an intervening region (IVR), and 6 kelch repeats that make up the β-propeller region that interacts with NRF2 (Fig. 1). Under basal conditions, a Keap1 homodimer binds to a single molecule of NRF2 at the 79ETGE82 [4] (hinge; strong affinity) and 29DLG31 [5] (latch; weak affinity) motifs in the Neh2 domain (Fig. 1). The Cul3-Rbx1 E3 ubiquitin ligase complex is then recruited to the α-helical region between these two motifs where it ubiquitylates NRF2 at seven lysine residues, targeting it for p97-mediated proteasomal degradation [[6], [7], [8], [9]]. Thus, under basal, non-stressed conditions, this complex is the principal mechanism by which NRF2 is degraded, resulting in a basal half-life of less than 30 min [10].

Mentioned above, NRF2 activation was initially characterized in the context of responding to xenobiotic-induced oxidative stress. Specifically, induction of NRF2 was originally shown to increase transcription of a variety of phase II/antioxidant-based genes designed to resolve the increase in reactive species and restore proper redox balance to the cell [[11], [12], [13]]. Critically, during this increased oxidative stress, Keap1-dependent degradation of NRF2 is disrupted via oxidative/electrophilic modification of several key cysteine residues (i.e., C151, C257, C273, C288, and C613) in Keap1 [14,15], allowing newly synthesized NRF2 to translocate to the nucleus, where it binds small MAF proteins (F/G/K) and initiates transcription of ARE-containing genes [11]. This mode of activation is known as the “canonical” pathway, as it reflects the most common transient mechanism by which NRF2 can be activated to resolve an acute oxidative stressor. Similar to oxidative modification, somatic mutations, competitive binding partners (i.e., other ETGE-containing proteins), as well as post-translational modification of Keap1 or NRF2 itself (i.e., phosphorylation, acetylation, and OGlcNAcylation), have all been shown to affect the ability of Keap1 to degrade NRF2 (summarized in Fig. 1)(Reviewed in Refs. [16,17]). Accordingly, it is important to note that a prolonged inability of Keap1 to degrade NRF2 leads to constitutive activation of NRF2 signaling, which as we will discuss in greater detail below, is generally associated with maladaptive consequences. This is evidenced by the well-established involvement of *KEAP1* mutations and chronic NRF2 activation in driving tumor progression and resistance [18,19], as well as the fact that whole body knock out of *Keap1* in mice leads to hyperkeratosis of the esophagus and death at one day of age [20]. Thus, Keap1-dependent degradation of NRF2 clearly represents a critical nexus of the switch from protective to pro-pathogenic activation of the NRF2 pathway.

#### 2.2.2 GSK3-SCF-β-TRCP

Along with Keap1-Cul3-Rbx1, another well-established regulator of NRF2 at the protein level is the SCF-β-TRCP E3 complex. However, unlike Keap1, which primarily governs NRF2 activation via redox sensing, β-TRCP degradation of NRF2 is mediated by GSK3-dependent phosphorylation. In a series of studies starting in 2006, Antonio Cuadrado and John Hayes’ groups identified that SCF-β-TRCP recruitment to NRF2 is activated by PI3K-AKT-GSK3-dependent phosphorylation of a DSGIS motif in the Neh6 domain (Fig. 1) [[21], [22], [23]]. β-TRCP interacts with NRF2 via the ^343^D(p)SGI(p)S^347^ motif, as well as a second interaction motif, ^382^DSAPGS^387^, although binding with the latter is not dictated by phosphorylation of the serine residues. The discovery that the PI3K/AKT/GSK3 axis regulates NRF2 protein levels opened a new realm of possible means of activation, particularly with regards to metabolic stress, growth factors, and pharmacological modulation of these critical kinases [24]. Much like Keap1, inhibition or induction of this cascade has been demonstrated to have a profound effect on NRF2 activation across different pathological settings, including acute liver injury [25], diabetic retinopathy [26], pancreatic, lung, and breast cancer [[27], [28], [29]], hepatic steatosis [30], ischemia-reperfusion injury [31], and several neurological disorders [5,32,33]. There is also the developing notion in the field that this mechanism of degradation compensates for loss of Keap1 function [34], although this has primarily been shown in models where Keap1 is genetically ablated. Importantly, β-TRCP can localize to both the cytosol and the nucleus, leading to the hypothesis that it can promote nuclear degradation of NRF2, as Keap1-dependent degradation occurs in the cytosol [24]. This is supported by literature showing NRF1 is degraded in the nucleus by β-TRCP [35], although this has yet to be proven with NRF2. Overall, the GSK3-SCF-β-TRCP axis clearly plays an important role in mediating NRF2 under certain pathophysiological contexts, including when Keap1 is non-functional.

#### 2.2.3 HRD1

The final E3 ligase shown to negatively regulate NRF2 at the protein level is the endoplasmic reticulum (ER)-associated ligase HRD1/synoviolin. While the primary function of HRD1 is to mediate ER-associated degradation (ERAD)-dependent ubiquitylation and proteasomal degradation of misfolded proteins, the Zhang lab showed in 2014 that HRD1 can translocate from the ER to the cytosol to target NRF2 for degradation, suppressing its activation during carbon tetrachloride-induced hepatic injury [36]. This same study identified that the C-terminus of HRD1 interacts with the Neh4-5 domains of NRF2 (Fig. 1), with a later group determining that the 125QSLVDPI131 motif in Neh4 is the specific binding site [37]. Importantly, HRD1 suppression of NRF2 seems to play an important role in several pathologies where chronic inflammation and ER stress are primary contributors, including liver cirrhosis, diabetic nephropathy, ischemic injury, and osteoporosis [[36], [37], [38], [39]]. This further supports the overlapping, but differential role of the three E3 ligase complexes, each of which can target NRF2 for degradation, but also respond to different stressors to regulate NRF2 activation or suppression depending on the nature and severity of the insult. This also indicates that there are multiple degradation mechanisms that can be targeted to activate NRF2 regardless of the pathological context, providing a broader scope of pharmacological targets for pathway activation or inhibition.

### 2.3 Modes of activation

#### 2.3.1 Canonical pathway

**Fig. 2:**
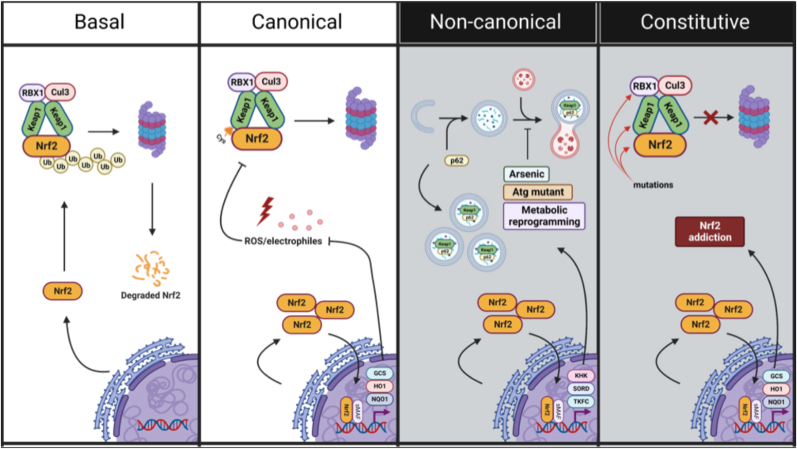
**Modes of NRF2 activation.** Nrf2 is regulated through multiple mechanisms. Under basal conditions, Keap1 binds NRF2 and targets it for Cul3-RBX1–mediated ubiquitination and proteasomal degradation, maintaining low transcriptional activity. Canonical activation occurs when oxidative or electrophilic stress inhibits Keap1-mediated degradation, allowing Nrf2 to accumulate, translocate to the nucleus, and induce cytoprotective targets such as GCS, HO1, and NQO1. Non-canonical activation involves alternative pathways, including p62 accumulation, *ATG* gene mutations, arsenic exposure, or metabolic reprogramming, which stabilize NRF2 independently of oxidative stress and promote selective gene expression, including metabolic enzymes. Constitutive activation arises from mutations in Keap1 or NRF2 that prevent ubiquitination, leading to persistent NRF2 nuclear localization, sustained target gene expression, and “NRF2 addiction.”

Discussed briefly above, canonical activation of NRF2 involves oxidative or electrophilic modification of reactive cysteines in Keap1. Cysteine modification results in a conformational change to the Keap1-Cul3-Rbx1-NRF2 complex that prevents ubiquitylation and traps bound NRF2, allowing newly synthesized NRF2 to escape Keap1-mediated proteasomal degradation and translocate to the nucleus to initiate target gene transcription (Fig. 2). Importantly, Keap1 is considered a cysteine-rich “redox sensor”, with ∼4 % of its total amino acid content being comprised of cysteine residues (27/624 in human; 25/624 in mouse). Discussed briefly above, the critical role of cysteine modification in driving NRF2 activation was initially identified in a series of studies done in the early 2000s. Among the many reactive cysteines identified (i.e., C151, C226, C257, C273, C288, C613, and C622/624), C151 represents the most commonly modified, as many of the best known pharmacological inducers of NRF2, including sulforaphane (SF), tertbutyl hydroquinone (tBHQ), dimethyl fumarate (DMF), and several bardoxolone analogs (CDDO-Me, CDDO-Im, CDDO-TFEA) all modify this key cysteine (Table 1) [14,15,[40], [41], [42], [43], [44], [45], [46], [47], [48], [49], [50], [51], [52], [53], [54], [55], [56], [57], [58], [59], [60]]. Contrastingly, other non-electrophilic modifiers, such as heavy metals, hydrogen peroxide, and the prostaglandin 15 d-PGJ_2_ target alternative cysteine residues [61]. This led to the notion that Keap1-dependent activation of NRF2 is governed by a “cysteine code”, with each cysteine reacting to its own subset or “class” of reactive agents [62]. Additionally, cysteine-independent activation of NRF2 can also be achieved via NRF2-Keap1 protein-protein interaction inhibitors (PPIs), which consist of a wide variety of peptides and small molecules that physically disrupt NRF2-Keap1 binding (i.e., SRS-5, Cpd 15, Cpd 7, AN-465/144,580, and 3-(Pyridin-3-ylsulfonyl)-5-(trifluoromethyl)-2H-chromen-2-one). While PPIs are generally considered to be a more favorable pharmacological approach over less-specific, cysteine-targeting electrophilic compounds, optimization is still underway to improve permeability and stability, as well as determine the *in vivo* therapeutic tolerability and efficacy of this promising class of compounds (reviewed in Ref. [63]).

**Table 1:**
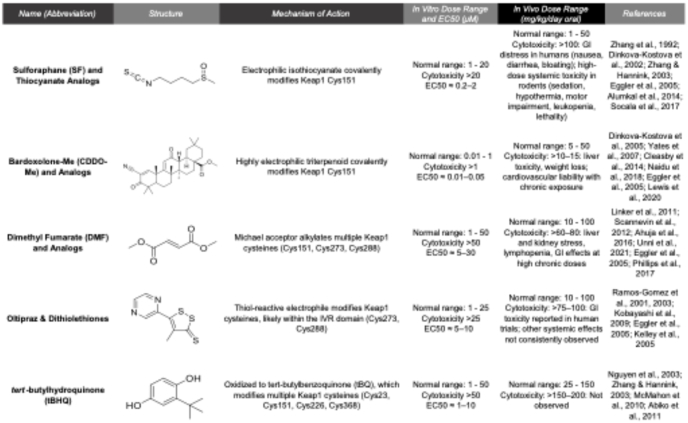
In vitro and in vivo dose ranges of classical NRF2 inducers and their mechanism of action.

Importantly, the same electrophilic reactivity that underlies robust NRF2 activation also confers limited molecular specificity. Critically, many canonical NRF2 inducers are capable of covalently modifying reactive cysteines on proteins beyond KEAP1, including metabolic enzymes, mitochondrial proteins, redox sensors, and signaling kinases, leading to perturbations in proteostasis, mitochondrial function, inflammatory signaling, and cellular redox balance at higher doses [64]. These off-target effects likely contribute to the dose-limiting toxicities observed *in vitro* and *in vivo*, including general cytotoxicity, hepatotoxicity, gastrointestinal irritation, immune modulation, and cardiovascular liability, as exemplified by clinical trials with bardoxolone and dimethyl fumarate [[65], [66], [67]]. While dietary electrophiles such as sulforaphane generally exhibit favorable safety profiles at nutritionally relevant exposures, supraphysiologic dosing or chronic administration could similarly engage non-selective thiol chemistry. Consequently, as indicated above, there is increasing emphasis on developing non-electrophilic NRF2 PPI-based modulators, as well as more targeted delivery strategies, prodrugs that require further metabolism, and medicinal chemistry-based modification of existing molecules, to improve selectivity and enhance therapeutic value.

#### 2.3.2 Non-canonical pathway

As the identification and characterization of compounds and drugs that could induce NRF2 via the canonical pathway continued to emerge, a new pathological mode of NRF2 activation was discovered. This discovery came with a critical realization in the field that NRF2 activation in a pathological setting was not always ideal, as its protective function could be hijacked by cancer cells to promote tumor progression. In 2010, five separate groups discovered “non-canonical” activation of NRF2, which typically occurs in the presence of autophagy dysfunction [[68], [69], [70], [71], [72]]. Specifically, mutations in key autophagy genes (i.e., *ATG5* and *ATG7*), or prolonged exposure to environmental toxicants (i.e., arsenic) resulted in chronic autophagy dysfunction and accumulation of the autophagy cargo adaptor SQSTM1/p62. Critically, p62 has a^349^DPSTGE^354^ motif, with phosphorylation of the serine resulting in preferential binding of p62 to Keap1, preventing its interaction with ETGE-containing NRF2 [68,69]. This p62-dependent sequestration of Keap1 results in prolonged activation of NRF2 downstream cascades, including those not normally turned on in an acute setting, which many studies have now shown is a primary driver of the metabolic reprogramming observed in certain cancers and arsenic-promoted pathologies [69,[73], [74], [75], [76], [77], [78], [79]](Fig. 2).

#### 2.3.3 Constitutive activation

Like non-canonical activation, somatic mutations or copy number alterations in *NFE2L2*/NRF2, *KEAP1*, *CUL3*, or *RBX1* have been reported to drive constitutive activation of NRF2 across a variety of cancer types, including lung, breast, head and neck, gall bladder, kidney, ovarian, and endometrial [4,18,[80], [81], [82], [83], [84], [85]]. Mutations and copy number alterations in NRF2 and its degradation machinery are particularly prevalent in non-small cell and squamous cell lung cancers, being present in as many as 30 % of cases in the latter [[86], [87], [88], [89], [90]]. Typically, these genetic alterations prevent either Keap1-NRF2 binding, or the ability of the E3 degradation complex to properly form, resulting in accumulation of NRF2 and chronic activation of its downstream pathways. This led to the concept of “NRF2 addiction” in cancer, where cancer cells become reliant on the increased antioxidant capacity, DNA repair capability, and proteostatic stability that hyperactivation of NRF2 affords [91] (Fig. 2). In fact, NRF2 activation has been linked to all the classical hallmarks of cancer set forth by Hanahan and Weinberg, inferring that inhibiting NRF2 represents a viable strategy for cancer treatment regardless of the post-initiation stage [92]. Evidence supporting NRF2 activation for chemoprevention versus NRF2 inhibition for chemotherapy will be discussed later.

### 2.4 Transcriptional programs

#### 2.4.1 Redox balance

One of the most critical functions of NRF2 is its regulation of redox homeostasis, particularly in the presence of stressors that are oxidative in nature or increase the production of secondary radical species. Among the many systems the cell has in place to handle increased oxidative stress, glutathione represents the first line of defense, as it is present in millimolar concentrations in most tissues and acts as a critical cofactor for several antioxidant systems. Glutathione synthesis and metabolism are particularly reliant on NRF2, as the enzymes responsible for generating and reducing glutathione are encoded by NRF2 target genes. This includes the catalytic and modifier subunits (*GCLC/GCLM*) of glutamate-cysteine ligase (GCS), which catalyzes the addition of glutamate to l-cysteine, as well as glutathione synthetase (*GSS*), which adds the glycine residue to complete the glutathione tripeptide [12,[93], [94], [95], [96], [97]]. Even the import of cystine, the precursor to cysteine, is governed by NRF2, as *SLC7A11*, a subunit of the system xc-glutamate-cystine antiporter, is a target gene [98]. Critically, the reduction of oxidized glutathione (GSSG) back to its reduced form (GSH) is also mediated by NRF2 target glutathione reductase (*GSR*) [99,100], and four of the critical GSH/NADPH-utilizing peroxide reduction systems, including glutathione peroxidase 2 (*GPX2*), sulfiredoxin 1 (*SRXN1*), thioredoxin reductase 1/thioredoxin (*TXNRD1, TXN*), and peroxiredoxins 1 and 6 (*PRDX1/6*) are directly regulated by NRF2 [99,[101], [102], [103], [104], [105], [106]]. Along with the enzymes that utilize NAPDH and GSH to reduce reactive species, most of the enzymes responsible for regenerating NAPDH, namely glucose-6-phosphate dehydrogenase (*G6PD)* of the pentose phosphate pathway, and malic enzyme 1 (*ME1*) and isocitrate dehydrogenase 1 (*IDH1*) of the citric acid cycle are also target genes [107]. Overall, it is easy to see why NRF2 was initially identified as the master regulator of redox homeostasis, as without it, reduction of reactive species is severely impaired.

Along with GSH synthesis and peroxide reduction, NRF2 also transcriptionally regulates several enzymes involved in the three phases of xenobiotic/drug metabolism. This includes phase I enzymes, i.e., cytochrome P450, family 2, subfamily a, polypeptide 5/6 (*CYP2A5*/*6*), carboxylesterase I (*CES1*), and aldo-ketoreductase family C1, members 1–3 (*AKR1C1-3*), phase II conjugation enzymes, i.e., glutathione-S-transferases alpha 1 and pi 1 (*GSTA1/GSTP1*) and NADPH quinone dehydrogenase 1 (*NQO1*), as well as phase III membrane transporters, i.e., ATP binding cassette subfamily C members 1–4 (*ABCC1-4*) [11,[108], [109], [110], [111], [112], [113], [114], [115], [116]]. Clearly, NRF2 plays an integral role in mediating many of the key redox-dependent reactions that govern the cellular response to oxidative or xenobiotic insult, including certain drugs/pharmacological compounds, which will be important in its mediation of chemoresistance, discussed later.

#### 2.4.2 Metabolism

Along with maintaining cellular redox status, NRF2 also plays a role in mediating several other key aspects of intermediary metabolism. For example, NRF2 regulation of lipid metabolism extends beyond just reduction of lipid peroxides, and many of the enzymatic regulators of lipid catabolism have been linked to NRF2, although identification of functional AREs is still required. This includes several lipases [phospholipase A2, group VII (*PLA2G7*) and lipase H (*LIPH*)], as well as acyl-CoA-oxidases 1 and 2 (*ACOX1/2*), critical drivers of peroxisomal fatty acid β-oxidation, which were shown to be regulated by SKN-1 the homolog of Nrf2 in *C. elegans* [117,118]. NRF2 regulation of lipogenesis in mice has been reported by several groups with contradictory results. While NRF2 binding to the promoter of nuclear receptor subfamily 0, group B, member 2/small heterodimer partner (*NR0B2*/SHP) has been reported, with NRF2 loss resulting in decreased hepatic expression, and induction the inverse, of several pro-lipogenic transcripts (*SREBP*, *FASN*, *PPARG*, and *SCD1*) [119], NRF2 ablation has also been inversely correlated with the expression of these same genes in the mouse liver [120,121]. Part of this discrepancy might be the result of diet, as a positive correlation was reported in mice fed a normal chow diet, and an inverse correlation was observed with mice fed a high fat or methionine/choline-deficient diet. Regardless, ARE validation and a more thorough characterization of NRF2 regulation of lipogenesis, particularly outside of a liver context, are still needed.

Recently, NRF2 regulation of different facets of carbohydrate metabolism has also begun to emerge. Mentioned above, G6PD, IDH1/2, and ME1 are all NRF2 targets that have been shown to process downstream metabolites of glucose to regenerate NAPDH. Three key enzymes involved in the non-oxidative branch of the PPP, namely 6-phosphogluconate dehydrogenase (*PGD*), transaldolase 1 (*TALDO1*), and transketolase (*TKT*) were also originally identified as direct transcriptional targets of NRF2 in NRF2 addicted lung cancer cells [107]. Glycogen break down can also be regulated by NRF2, as two key glycogenolytic enzymes, glycogen branching enzyme (*Gbe1*) and muscle-type PhKα subunit (*Phka1*), were identified as NRF2 target genes in the skeletal muscle of muscle-specific *Keap1*^*−/−*^ mice [122]. Recent work from the Zhang group also identified four novel target genes in arsenic-exposed mouse liver, including three involved in the polyol pathway [ketohexokinase (*Khk*), sorbitol dehydrogenase (*Sord*), and triokinase/FMN cyclase (*Tkfc*)], and one regulator of gluconeogenesis [hepatocyte nuclear factor 4 (*Hnf4A*)] [79]. Overall, it appears that NRF2 modulation of carbohydrate metabolism primarily arises during a chronic, constitutive, or non-canonical activation setting, and verification of the relevance of this NRF2-regulated axis across additional tissue types and pathological contexts warrants further investigation.

Finally, another major metabolic hub regulated by NRF2 is iron homeostasis. Proper import, export, storage, and incorporation of iron into iron-containing proteins is indispensable not only in driving mitochondrial metabolism and oxygen transport but also preventing the formation of harmful Fenton-derived free radical species. A critical cofactor for iron incorporation into proteins is the porphyrin ring heme. Both heme synthesis and heme degradation are controlled, in part, by NRF2. For synthesis, the mitochondrial heme transporter ATP binding cassette subfamily B member 6 (*ABCB6*) and ferrochelatase (*FECH*), which incorporates ferrous iron into the core of the heme ring in the final step of heme synthesis, both have validated AREs [[123], [124], [125]]. For degradation, the heme catabolizing enzymes alpha-1-microglobulin/bikunin precursor (*AMBP*), heme oxygenase I (*HMOX1*), and biliverdin reductase B (*BLVRB*) are also verified NRF2 target genes [[124], [125], [126], [127], [128]]. Following its import by transferrin, iron can be stored in the critical iron storage protein ferritin, whose light (*FTL*) and heavy (*FTH1*) chains are encoded by NRF2 target genes [124,129,130]. Iron that is not incorporated into proteins or stored in ferritin cages contributes to the labile iron pool, with excess ferrous iron getting exported by NRF2-regulated ferroportin-1 (*SLC40A1*/FPN1) [125,131]. Finally, the Zhang lab recently discovered that the E3 ligase HECT and RLD domain containing E3 ubiquitin protein ligase 2 (*HERC2*), which among its many roles, targets F-box and leucine rich repeat protein 5 (FBXL5), a key mediator of iron regulatory 1/2 protein levels (IRP1/2), for proteasomal degradation, is also an NRF2 target [132]. Importantly, dysregulation of NRF2-dependent regulation of iron signaling has significant consequences, as iron accumulation results in an iron-dependent mode of cell death termed ferroptosis [133]. As such, tight control of iron homeostasis by NRF2 is critical for cell survival, identifying this axis as a targetable vulnerability in chemoresistant, NRF2 addicted cancers [[134], [135], [136], [137]].

#### 2.4.3 Protein homeostasis

Perhaps one of the more intriguing and less established branches of cellular homeostasis recently linked to NRF2 is proteostasis. While fellow CNC family member NRF1 is better known for its regulation of the expression of a host of proteasomal subunits, facilitating proteasome assembly during proteasomal inhibition [138,139], some proteasome subunits and associated factors have been shown to be mediated by NRF2. This includes proteasome subunits α1 and β5 (*PSMA1/PSMB5*), and the proteasome maturation protein (*POMP*) [[140], [141], [142]]. Another critical protein quality control pathway linked to NRF2 is the autophagy-lysosome pathway, and the autophagy cargo adaptor sequestosome 1 (*SQSTM1*/p62), initiation proteins autophagy related 5/7 (*ATG5/7*) and unc-51 like autophagy activating kinase 1 (*ULK1*), as well as lysosome associated membrane protein 2 (*LAMP2*), a critical player in chaperone mediated autophagy, all have functional AREs [143,144]. Lastly, protein folding, and stability can also be partially controlled by NRF2, as expression of the key protein quality control transcription factor heat shock factor 1 (*HSF1*) and the segregase p97 (*VCP*/p97) are mediated by NRF2, particularly in the context of arsenic exposure [145,146]. Much like its governance of metabolic homeostasis, NRF2 regulation of proteostasis has only been demonstrated across a limited number of contexts, with a more extensive validation of its pan-context relevance still needed. However, what has become increasingly clear as the number of NRF2-mediated transcriptional programs has grown, is that while acute activation seems to favor antioxidant/detoxification pathways, chronic induction results in the activation of alternative, less redox-centric cascades.

### 2.5 Crosstalk and alternative modes of regulation

**Fig. 3:**
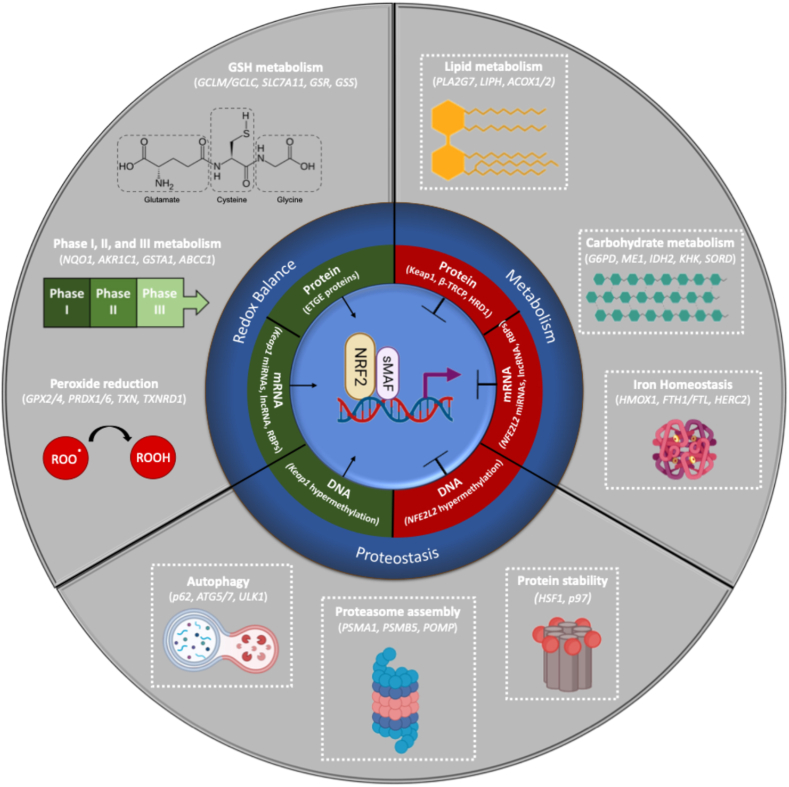
**NRF2 transcriptional programs and regulatory mechanisms.** Schematic diagram indicating the diverse cellular processes governed by NRF2 and the mechanisms controlling its activity. The central circle depicts NRF2 binding to DNA and activating transcription of target genes, with regulatory influences including its E3 degradation machinery (e.g., KEAP1, β-TRCP, HRD1), mRNA regulation (miRNAs, lncRNAs, RBPs), and DNA hypermethylation. Surrounding the core circle are NRF2-driven transcriptional programs, divided into three functional categories: **Redox balance** (glutathione metabolism, phase I-III metabolism, peroxide reduction), **Metabolism** (lipid, carbohydrate metabolism, iron homeostasis), and **Proteostasis** (autophagy, proteasome assembly, protein stability). Key representative genes for each pathway are indicated in parentheses. Boxed pathways represent NRF2-regulated programs supported by verified AREs whose physiological relevance are context-dependent and secondary to its canonical redox and detoxification functions.

Despite being primarily regulated at the protein level, NRF2 expression can also be controlled at the DNA and mRNA levels. At the DNA level, it is well documented that epigenetics can influence *NFE2L2*/NRF2 expression across several cancer types, as hypermethylation of the *KEAP1* promoter and hypomethylation of the *NFE2L2*/NRF2 promoter can lead to increased expression of NRF2 [[147], [148], [149], [150], [151], [152]]. In the case of mRNA regulation, *NFE2L2* mRNA stability is influenced by several non-coding RNAs, including numerous microRNAs (i.e., *miR 28, 34a, 93, 144,* and *146a*) and long non-coding RNAs (i.e., *UCA1* and *MEG3*) that increase or decrease expression depending on the binding site (3′ vs. 5’ UTR) [[153], [154], [155], [156], [157], [158], [159]]. Conversely, miRNAs and lncRNAs that target *KEAP1* transcript to enhance NRF2 expression have also been reported (*miR125b*, *miR200a*, and *MALAT1*) [[160], [161], [162]]. It is also worth mentioning that at least three RNA binding proteins have been identified that enhance *NFE2L2*/NRF2 mRNA stability, AU-rich binding factor 1 (AUF1), human antigen R (HuR), and far upstream element binding protein 1 (FUBP1) stabilizing *NFE2L2* mRNA, and one, RNA binding motif protein 47 (RBM47), that decreases expression via promoting the stability of *KEAP1* and *CUL3* transcripts [[163], [164], [165]]. Finally, as mentioned earlier, NRF2 activity has been linked to several other major signaling nodes, including NF-κB, mTOR, Notch, and AhR, with the latter two transcription factors having response elements present in the *NFE2L2*/NRF2 promoter [166,167]. The complex interconnectivity of NRF2, including multiple modes of regulation, a diversity of downstream transcriptional programs, and crosstalk with other integral signaling cascades, make it easy to see why its relevance in disease continues to grow. Additionally, while this complexity introduces an added layer of difficulty to understanding the nature of NRF2 signaling in any given pathological context, it also provides multiple therapeutic avenues to target this critical transcription factor in disease. A summary of the negative and positive regulators of NRF2, as well as the different well-established versus newly emerging transcriptional programs it regulates, is provided in Fig. 3.

## 3 NRF2 in disease prevention

Since the seminal discovery that NRF2 binds AREs to drive cytoprotective gene expression, extensive work has revealed the broad scope of its influence across nearly every physiological response. Accordingly, the pathological contexts in which NRF2 plays a role are equally diverse, encompassing cancer, diabetes, neurodegeneration, cardiovascular and pulmonary diseases, and chronic inflammatory states. In each case, the consequences of NRF2 activation are highly context dependent, i.e., transient and appropriately regulated activation is generally cytoprotective, whereas sustained or dysregulated activation contributes to disease progression. Below, we will briefly discuss the protective role of NRF2 in mitigating disease onset and progression, then transition into inhibiting NRF2 for cancer prevention, which has been a more recent focal point in the NRF2 field.

### 3.1 Chemoprevention

Transient and controlled activation of NRF2 is now firmly established as one of the most robust endogenous defenses against carcinogenesis. Outlined in detail above, foundational studies in the late 1990s and early 2000s clearly demonstrated that NRF2 orchestrates inducible expression of a suite of cytoprotective antioxidant and phase II detoxification enzymes, including NQO1, GSTA1, GCS, HMOX1, and AKR family members. Along these lines, several pivotal *in vivo* studies established that NRF2 activation provides robust protection against carcinogen-induced tissue injury, oxidative DNA damage, and mutagenicity. For example, pharmacological induction of NRF2 with agents such as oltipraz or dithiolethiones was shown to enhance phase II enzyme expression, reduce DNA adduct formation, and mitigate oxidative damage in wild-type mice exposed to various carcinogens, including benzo[a]pyrene, nitrosamines, and polycyclic aromatic hydrocarbons (PAHs). Importantly, all protective phenotypes were dramatically reduced or lost in *Nrf2*^*−/−*^ animals, which exhibited increased tumor burden and loss of chemotherapeutic efficacy [54,55,[168], [169], [170]]. These complementary genetic and pharmacologic studies provided definitive seminal evidence that NRF2 is a central regulator of cellular defense mechanisms that mitigate carcinogenic stress, governing the detoxification, antioxidant, and cytoprotective pathways that prevent tumor initiation. Building on this foundation, a large body of research from 2005 to present has continued to validate that pharmacological activation of NRF2 is sufficient to protect against tumor initiation across multiple organ systems. This includes both dietary and synthetic NRF2 activators, such as sulforaphane, curcumin, CDDO-Im, and CDDO-Me (bardoxolone), which were shown to consistently reduced tumor burden in models of lung, liver, stomach, and skin carcinogenesis [[171], [172], [173], [174]]. Collectively, almost three decades of NRF2-based research have converged to generate a central conclusion: short-term, context-appropriate activation of NRF2 is a critical frontline defense against the initiation of carcinogenesis, explaining why NRF2 remains a major therapeutic target for chemoprevention.

### 3.2 NRF2 in other pathologies

Since NRF2 functions as a central regulator of cellular defense across all tissues, it is not surprising its protective role has been implicated in disease states beyond cancer. In the context of diabetes, NRF2 is critical for maintaining pancreatic β-cell integrity, which are inherently vulnerable to oxidative and nitrosative stress due to low antioxidant capacity, at least in rodents [175]. Activation of NRF2 preserves β-cell function, supports insulin secretion, and mitigates the onset of hyperglycemia in preclinical models, whereas NRF2 deficiency exacerbates β-cell dysfunction and glucose intolerance [[176], [177], [178]]. Outside the pancreas, NRF2 modulates hepatic and adipose metabolism, reducing lipotoxicity, steatosis, obesity, oxidative stress, and inflammatory signaling, though chronic or excessive activation can perturb insulin signaling and increase hepatic glucose production [79,[179], [180], [181], [182], [183], [184]]. In the brain, neurons and glia are similarly dependent on NRF2-mediated redox homeostasis, as they are highly sensitive to mitochondrial dysfunction and oxidative damage. Activation of NRF2 in neuronal and astrocyte populations enhances antioxidant capacity, attenuates neuroinflammation, and promotes mitochondrial biogenesis and autophagic clearance of damaged organelles and aggregated proteins, thereby improving survival in models of Alzheimer's and Parkinson's disease, as well as Amyotrophic lateral sclerosis [143,[185], [186], [187], [188], [189], [190]]. These findings underscore NRF2 as a promising neuroprotective target, though therapeutic strategies must achieve adequate blood-brain-barrier penetration and cell-type specificity to avoid systemic off-target effects. Finally, NRF2's protective scope extends beyond metabolic and neurodegenerative disorders to injury and stress models as well. For example, *Nrf2*^*−/−*^ mice exhibit increased susceptibility to chemical hepatotoxins, pulmonary oxidants, and ischemia-reperfusion injury, whereas pharmacologic activation or genetic stabilization of NRF2 enhances tissue recovery, reduces inflammation, and promotes cytoprotection [[191], [192], [193], [194], [195], [196], [197]]. Collectively, these studies position NRF2 as a universal sensor–effector molecule, capable of orchestrating antioxidant, anti-inflammatory, and adaptive metabolic responses to preserve cell and tissue homeostasis under diverse pathological conditions. Importantly, the benefits of NRF2 activation are context-dependent, and the need for more precise temporal and tissue-specific regulation to maximize therapeutic efficacy while minimizing potential deleterious effects persists.

### 3.3 Therapeutic implications

Given its broad protective scope, pharmacological modulation of NRF2 remains an area of active translational investigation. Highlighted above, controlled activation of NRF2 shows promise in chemoprevention, as well as the treatment of chronic inflammatory, metabolic, and neurodegenerative conditions. Despite the identification of potent NRF2 activators such as bardoxolone methyl and sulforaphane, clinical translation has been hampered by off-target effects and context-dependent toxicity. As such, continued elucidation of NRF2's regulation, now spanning epigenetic, transcriptional, and post-translational mechanisms, will be essential for the development of therapeutics that exploit its protective potential while minimizing adverse outcomes. Collectively, these findings highlight NRF2 as both a molecular safeguard and a potential liability. Its role as a master regulator of the cellular stress response is clear, but its context-specific regulation and lack of target-specific inducers underscores the importance of precision targeting. While the emergence of NRF2 and KEAP1-specific PPIs has enhanced the prospect of more specifically targeting this pathway in a less toxic manner, significant work still needs to be done to transition these compounds to clinic. Regardless, as our understanding of its regulation and downstream effectors continues to advance, the therapeutic window for NRF2 modulation will become increasingly well defined, moving this pathway from experimental promise to clinical utility.

## 4 NRF2 in disease intervention

### 4.1 Chemotherapy

The phenomenon of NRF2 induction and its relationship to disease prevention has been well-documented since its official discovery in 1994. Until 2008, NRF2 was primarily recognized for its cytoprotective role, frequently referred to as a “beneficial” cellular component. This notion was due to the relatively recent emergence of the NRF2 field, where, as discussed above, early investigations of NRF2 signaling identified downstream effectors associated with various cytoprotective responses involved in the prevention of disease. However, despite substantial evidence suggesting NRF2's solely protective capacity, the notion that NRF2's influence on various pathways could be exploited to initiate and even progress certain diseases was an important, often overlooked area of study. This question represented a significant knowledge gap in the NRF2 field for 20 years until the Zhang lab, in 2008, revealed the “dark side” of NRF2 signaling in the context of cancer [198]. This critical early work indicated that various aggressive and resistant cancer subtypes, particularly non-small cell lung carcinomas, exhibited a “NRF2-high” phenotype. This turned out to be due to gain-of-function mutations in the Neh2 domain of *NFE2L2*/NRF2 or loss-of-function mutations in the Kelch domain of *KEAP1* that destabilize the NRF2-KEAP1 interaction and promote NRF2 accumulation [198]. This finding significantly shifted the NRF2 field's paradigm, as it was one of the first pieces of evidence to challenge the widely accepted hypothesis that NRF2 is exclusively cytoprotective. Various studies from other groups confirmed this phenomenon, and the term “dark side” of NRF2 was officially coined. This not only filled a significant knowledge gap in the NRF2 field but also demonstrated the therapeutic potential of inhibiting NRF2 in resistant, NRF2-high cancers like lung cancer, the deadliest form of cancer in 2023 [199]. While NRF2 inhibition was initially elicited through genetic modification, the idea of replicating the chemosensitive phenotype using a small-molecule inhibitor of NRF2 emerged as an attractive prospect for researchers worldwide. However, identifying an NRF2-specific inhibitor using traditional high-throughput screening methods presented a substantial challenge.

The first NRF2 inhibitor identified was a molecule called brusatol. Brusatol is a natural product extracted from *Brucea javanica*, a shrub native to Southeast Asia and Northern Australia. Using a high-throughput MDA-MB-231-ARE-Luciferase assay to identify molecules that reduce ARE-luciferase activity, brusatol demonstrated a clear dose-response with an IC50 ≤ 80 nM [200]. Additionally, it was shown to decrease NRF2 and downstream target gene levels through western blot and qRT-PCR analyses. Importantly, using an A549 lung xenograft model, the combination of brusatol and cisplatin increased cytotoxicity, reduced tumor size, and decreased tumor score compared to vehicle or each individual molecule alone [200]. However, concerns about toxicity following its administration surfaced since brusatol alone at doses higher than 80 nM in cell-based assays or 2 mg/kg in murine models caused severe toxicity. This raised a critical question: If NRF2 knockout in mice is nonlethal with no significant changes in phenotype, why does inhibiting NRF2 with Brusatol cause toxicity at such low doses?

This question was answered in 2017 when brusatol was discovered to be a global translation inhibitor, significantly affecting short-lived proteins, including NRF2, in the cell [201]. Despite the unfortunate reality of its non-specificity, brusatol is still employed in a laboratory setting to achieve pharmacological inhibition of NRF2. In addition, several groups focused on identifying brusatol's actual target, with PI3Kγ being the best candidate identified to date [202]. To decrease its toxicity, other researchers have sought to generate analogs with reduced off-target effects. For instance, one recent study identified a 3-β-homoalanine analog of brusatol that demonstrated significantly decreased toxicity in mice [203]. If this molecule proves entirely non-toxic at effective doses required for NRF2 inhibition, it has the potential to serve as a non-toxic, non-selective NRF2 inhibitor. Following the initial discovery of brusatol, laboratories worldwide adopted similar strategies to screen compounds that inhibit NRF2 transcriptional activity using ARE-luciferase systems or cell-viability assays. Molecules identified by this approach, such as halofuginone, malabaricone-A, trigonelline, and others have been described in (Table 2) [200,201,[203], [204], [205], [206], [207], [208], [209], [210], [211], [212], [213], [214], [215]]. Despite these molecules' potent inhibitory capacity on NRF2 activity and disease progression, they lack specificity, whether proven or unproven, to NRF2 itself. To overcome this issue, several groups developed novel strategies to identify molecules that selectively inhibit NRF2.

**Table 2:**
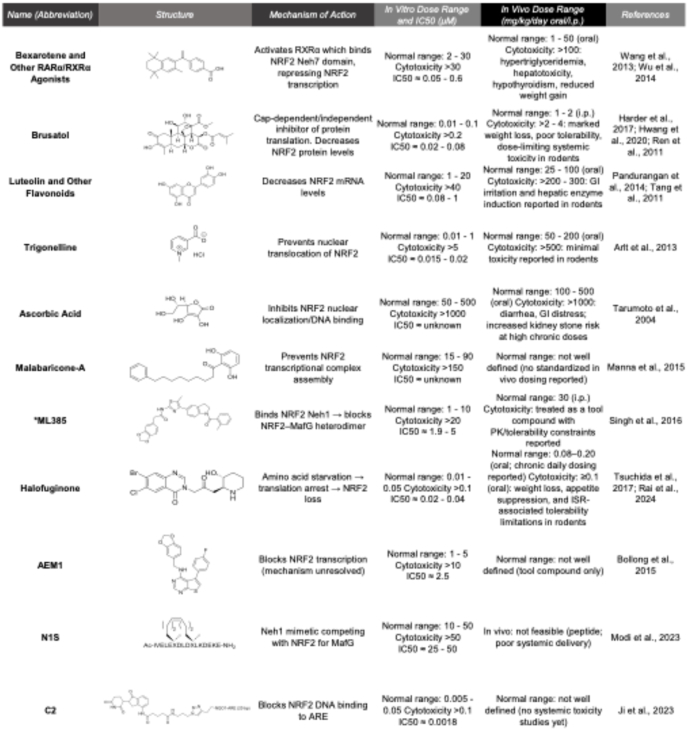
In vitro and in vivo dose ranges of NRF2 inhibitors and their proposed mechanism of action.

Previously, novel NRF2 inhibitors were screened using an ARE-luciferase system, which identifies molecules that decrease NRF2-dependent ARE-luciferase activity, a direct output of NRF2 transcriptional activity. However, this strategy often identifies translational inhibitors, as global protein synthesis inhibition would also result in decreased ARE-luc protein levels. The first reported targeted approach to NRF2 inhibition sought molecules that activate RXRα, as activated RXRα can interact with the Neh7 domain of NRF2, repressing its transcriptional activity [210,211,216]. Molecules such as retinoic acid, bexarotene, and other RXRα and RARα activators were found to promote NRF2 downregulation. However, due to RXRα activation's association with various cell signaling pathways, activation may lead to unwanted off-target effects [210,211,216]. Despite these concerns, this discovery initiated the development of NRF2 inhibitors using a targeted approach. Instead of focusing on the RXRα-Neh7 interaction, scientists began to target NRF2 regions essential for its activity, such as Neh1. Mentioned earlier, for NRF2 to function as a transcriptional regulator, it must first form a dimer with sMAF proteins to bind to AREs in the promoters of downstream target genes. NRF2 cannot form a functional homodimer and cannot bind to AREs as a single molecule. Supporting this notion, the Biswal group published the first in-class NRF2 inhibitor targeting the Neh1-sMaf protein-protein interaction in 2016 [217]. Through various experiments, they demonstrated that their molecule, ML-385, is an NRF2-selective molecule that directly binds to NRF2's Neh1 domain and prevents dimerization with sMAF proteins. They also provided translational data in relevant cell and murine models, showing that ML-385 works synergistically with cisplatin to mitigate cancer cell proliferation and tumorigenesis. However, issues with reproducibility have been reported by various labs, unable to replicate the original manuscript's results. Using a similar discovery strategy, the Parkinson lab published a molecule called N1S, a stapled peptide mimetic of the Neh1 domain of NRF2 [213]. However, while N1S's potential for inhibiting NRF2 is high in a biochemical setting, in cellular environments, a significant decrease in activity is observed, suggesting potential issues with cell permeability. Additionally, *in vivo* translation of this mimetic's functionality has yet to be demonstrated.

A newer approach to the targeted and selective inhibition of NRF2 involves the use of proteolysis targeting chimeras, also known as PROTACs. These heterobifunctional molecules function as catalytic degraders of a specific target protein, making them ideal for inhibiting ‘undruggable’ targets (i.e., transcription factors). PROTACs were originally intended to target proteins without a defined structure or binding pocket that a small molecule can be designed to occupy; due to NRF2's lack of structure or defined binding pockets, PROTAC development towards NRF2 degradation has emerged as an attractive drug discovery strategy. Therefore, NRF2 inhibition could be alternatively achieved through PROTAC-mediated degradation. Specifically, if a molecule can be identified that binds selectively and tightly to NRF2 itself, regardless of whether it affects NRF2's activity, that molecule could be engineered into a PROTAC. This is achieved by conjugating the molecule to an optimized linker that is bound to an E3 ligase binding ligand. The resulting PROTAC can simultaneously bind to NRF2 and the intended E3 ligase, leading to the ubiquitination and proteasomal degradation of the NRF2 protein. In 2023, a group published the first NRF2 PROTAC of its kind, using a synthetic ARE as the target-binding ligand. They successfully demonstrated that their molecule labelled as C2 could selectively degrade NRF2 or the NRF2/MAFG heterodimer [214]. C2 achieved a DC50 of 1.854 nM, demonstrating the therapeutic potential of this molecule to degrade the NRF2 protein efficiently at very low concentrations. In addition to addressing the previous issue of specificity, this molecule can also evade toxicity as low nanomolar concentrations are sufficient to target NRF2. However, because a 20 base-pair oligonucleotide is used as the target binding ligand, questions about formulation and ADME (Absorption, Distribution, Metabolism, and Excretion) arise. The process of formulating a molecule containing DNA could potentially pose challenges if an oral formulation ends up being developed.

### 4.2 Therapeutic implications

**Fig. 4:**
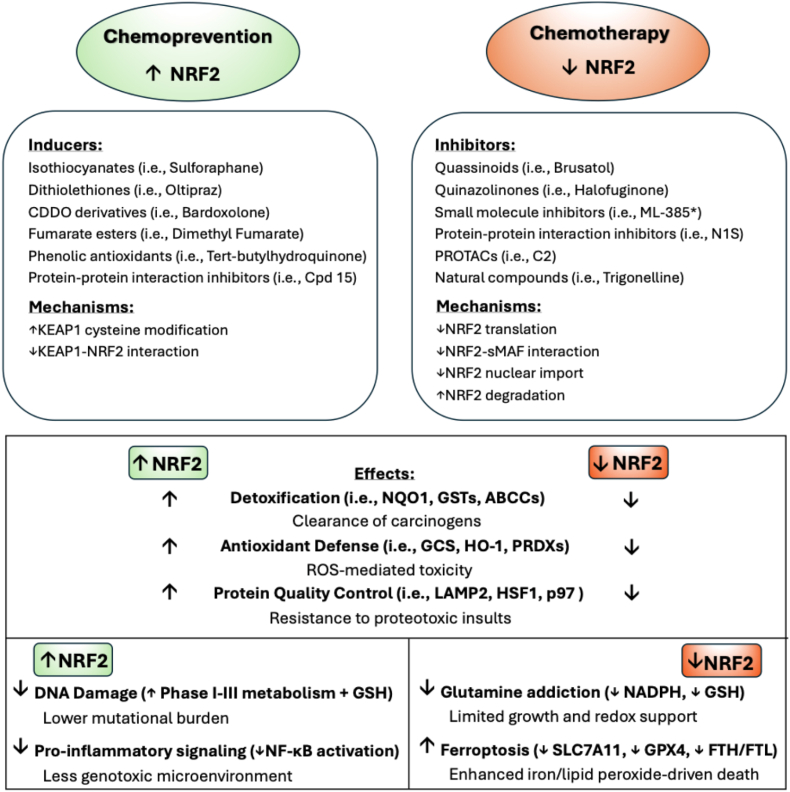
***Pharmacological modulation of NRF2 and its cancer-dependent effects.*** This figure summarizes known inducers (left, green) and inhibitors (right, red) of NRF2, alongside the mechanisms by which they regulate NRF2 activity. NRF2 inducers, including isothiocyanates, dithiolethiones, CDDO derivatives, fumarate esters, phenolic antioxidants, and protein-protein interaction inhibitors, primarily act by modifying KEAP1 cysteines resulting in a conformational change or disrupting the KEAP1-NRF2 interaction. NRF2 inhibitors, such as quassinoids, quinazolinones, small molecule inhibitors, protein-protein interaction inhibitors, PROTACs, and natural compounds, can reduce NRF2 translation, nuclear import, or its interaction with sMAF, or enhance its degradation. The downstream effects of NRF2 activation versus inhibition in a cancer context are highlighted: increased NRF2 promotes detoxification, antioxidant defense, protein quality control, reduced DNA damage, and suppression of pro-inflammatory signaling, supporting chemopreventive outcomes. Conversely, decreased NRF2 limits glutamine metabolism, enhances ferroptosis, and sensitizes cells to oxidative and proteotoxic stress, potentiating chemotherapeutic efficacy.

As our comprehension of NRF2's role in both preventing and propagating diseases deepens, strategies to impede its functionality have become increasingly refined. The identification of NRF2's ‘dark side’ has sparked an emphasis on the development of selective inhibitors, encompassing analogs of existing inhibitors and innovative small molecules. Numerous laboratories are now employing cutting-edge screening tactics, such as assays that test for molecules disrupting the Neh1-sMaf interaction, to pinpoint small molecules capable of specifically curbing NRF2's transcriptional activity while maintaining minimal off-target effects. Moreover, the use of PROTACs for the direct degradation of NRF2 has emerged as a promising new technology. Despite emerging challenges related to potency, reproducibility, and selectivity, the development of these targeted NRF2 inhibitors remains an exciting cornerstone for future disease prevention techniques and therapeutic strategies. The “light” versus “dark” side of NRF2 in cancer, including identified inducers and inhibitors, is summarized in Fig. 4.

Despite the identification of multiple small molecules capable of suppressing NRF2 activity, a consistent and recurring limitation across nearly all reported NRF2 inhibitors is the narrow or poorly defined therapeutic window (Table 2). In many cases, effective concentrations required for NRF2 inhibition overlap with doses that elicit off-target toxicity, raising fundamental concerns regarding translational feasibility. This limitation is particularly evident for compounds initially identified through ARE-luciferase or cell-based screening strategies, which frequently yield global protein translation inhibitors such as brusatol or halofuginone. While these agents potently suppress NRF2 protein levels, their mechanisms of action are inherently non-specific, leading to widespread depletion of short-lived proteins and dose-limiting systemic toxicity *in vivo*. Similarly, nuclear receptor-based approaches, including RXRα and RARα agonists such as bexarotene, achieve NRF2 repression through indirect transcriptional mechanisms that are associated with well-documented metabolic and endocrine liabilities, including hyperlipidemia, and thyroid dysfunction [[218], [219], [220]]. Although these agents demonstrate NRF2 inhibitory activity at clinically relevant doses, their extensive pleiotropic effects significantly constrain their utility as selective NRF2 inhibitors. In contrast, more recently developed targeted inhibitors, such as ML385 and the stapled peptide N1S, offer improved mechanistic specificity by directly disrupting NRF2–MAFG interactions. However, these molecules suffer from poor pharmacokinetic properties, limited cellular permeability, or inconsistent reproducibility across laboratories, further underscoring the challenges associated with targeting NRF2 directly. Collectively, these findings indicate that, to date, no small-molecule NRF2 inhibitor exhibits the combination of specificity, potency, and tolerability required for safe long-term systemic administration. Rather, most available compounds either achieve NRF2 suppression through global stress responses or require doses that approach or exceed toxicity thresholds *in vivo*. These limitations highlight a critical gap in the field and emphasize the need for next-generation strategies that decouple NRF2 inhibition from generalized cellular stress, including improved medicinal chemistry, targeted delivery systems, and emerging modalities such as PROTAC-based degraders.

## 5 Limitations and future directions

Overall, the growth that the NRF2 field has experienced over the past three decades has been substantial. This exponential increase in relevance indicates the key role that this master regulator plays in mediating multiple pathways across a variety of physiological and pathological contexts. However, despite an immensely evolved understanding of how activation or inhibition of NRF2 and its downstream cascades affects any given pathology, several critical hurdles in the field persist. Chief among these is translation of promising experimental therapeutics into viable drugs used in clinic. This pitfall stems from a variety of reasons, including the difficulty of targeting transcription factors to achieve specific inhibition, and the off-target toxic effects of the most potent activators. Despite this limitation, several NRF2 inducers have progressed to clinical trials, and many of the new approaches, including protein-protein interaction inhibitors, are beginning to show promising results (Table 1). While progress with regards to viable inducers has continued at a steady pace, attaining specific inhibition of NRF2 has remained a frustratingly difficult problem to address. Most inhibitors identified to date successfully inhibit NRF2, and have noticeable effects *in vitro* and *in vivo*, particularly in a cancer setting (Table 2). However, most of these compounds are non-specific, achieving their effects through altering protein translation, or other off-target toxicities that extend to pathways outside of the NRF2 cascade. Some recent workarounds have generated excitement in the field, including micelle-based delivery (i.e., halofuginone), medicinal chemistry-based modification of existing inhibitors, peptide-based mimetics that prevent the NRF2-MAFG interaction or DNA binding, PROTACs, and of course screening for more on-target, potent compounds. Thus, despite the seemingly endless elusiveness of obtaining an NRF2-specific inhibitor, current efforts have continued to make progress towards targeting NRF2 in a way that is less toxic to normal cells and tissues.

Mentioned briefly above, another critical need in the field is proper validation of “putative” NRF2 target genes. While many of the canonical target genes, most of which were identified in the early days of the field via microarray and ChIP-seq-based approaches, have been fully validated, many novel target genes still require this critical step. This can be evidenced by the growing notion in the field that NRF2 can affect the transcription of greater than 300 genes; however, whether this regulation is direct or indirect often still requires further clarification. This is particularly true in a chronic activation setting, as activated downstream cascades extend well beyond the bona fide antioxidant/detoxification target genes originally identified in an acute setting. Studies showing that NRF2 can regulate novel aspects of iron, lipid, and carbohydrate metabolism, as well as protein synthesis, stability, and turnover, have opened new avenues of NRF2-based discovery. Tissue-specific genetic modulation of NRF2 has also provided insight into its relevance in several diseases, including diabetes, liver disease, and heart disease, where global knockout left uncertainties about the primary versus secondary effects of the observed phenotypes. The emergence of CRISPR and other gene-editing technologies has also facilitated the discovery of NRF2-specific changes, as AREs can be point mutated to determine their functionality, or NRF2 or a critical downstream gene of interest, can both be deleted simultaneously to determine the specificity of the observed phenotype to NRF2. Similarly, the dramatic evolution of single-cell and bulk RNA-seq, quantitative proteomics, and metabolomic arrays for specific metabolite subsets have significantly enhanced our ability to categorize NRF2-specific signatures for a given cell/tissue-type, pathological context, or treatment of interest.

As the field has continued to expand and evolve, one thing has remained clear, NRF2 is a critical master regulator of cellular function and survival during stress. Critically, despite the vast expanse of NRF2-based literature that now exists, there is still plenty of room for growth and innovation in the field. This is particularly true in the context of NRF2-based therapeutics, which appear to be on the cusp of finally attaining clinical relevance. While much has been discovered, it will be exciting to see how this critical field continues to grow in the coming decades.

## CRediT authorship contribution statement

**Wei-tai Chen:** Conceptualization, Writing – original draft, Writing – review & editing. **Nicholas W. McKee:** Writing – original draft, Writing – review & editing. **Damaris Kuhnell:** Writing – original draft, Writing – review & editing. **Matthew Dodson:** Conceptualization, Supervision, Visualization, Writing – original draft, Writing – review & editing.

## Declaration of competing interest

The authors declare that they have no known competing financial interests or personal relationships that could have appeared to influence the work reported in this paper.

## Data Availability

No data was used for the research described in the article.
